# Monoester-Diterpene *Aconitum* Alkaloid Metabolism in Human Liver Microsomes: Predominant Role of CYP3A4 and CYP3A5

**DOI:** 10.1155/2013/941093

**Published:** 2013-06-23

**Authors:** Ling Ye, Xiao-Shan Yang, Lin-lin Lu, Wei-Ying Chen, Shan Zeng, Tong-Meng Yan, Ling-Na Dong, Xiao-Juan Peng, Jian Shi, Zhong-Qiu Liu

**Affiliations:** ^1^Department of Pharmaceutics, School of Pharmaceutical Sciences, Southern Medical University, Guangzhou, Guangdong 510515, China; ^2^International Institute for Translational Chinese Medicine, Guangzhou University of Chinese Medicine, Guangzhou, Guangdong 510006, China

## Abstract

*Aconitum*, widely used to treat rheumatoid arthritis for thousands of years, is a toxic herb that can frequently cause fatal cardiac poisoning. *Aconitum* toxicity could be decreased by properly hydrolyzing diester-diterpene alkaloids into monoester-diterpene alkaloids. Monoester-diterpene alkaloids, including benzoylaconine (BAC), benzoylmesaconine (BMA), and benzoylhypaconine (BHA), are the primary active and toxic constituents of processed *Aconitum*. Cytochrome P450 (CYP) enzymes protect the human body by functioning as the defense line that limits the invasion of toxicants. Our purposes were to identify the CYP metabolites of BAC, BMA, and BHA in human liver microsomes and to distinguish which isozymes are responsible for their metabolism through the use of chemical inhibitors, monoclonal antibodies, and cDNA-expressed CYP enzyme. High-resolution mass spectrometry was used to characterize the metabolites. A total of 7, 8, and 9 metabolites were detected for BAC, BMA, and BHA, respectively. The main metabolic pathways were demethylation, dehydrogenation, demethylation-dehydrogenation, hydroxylation and didemethylation, which produced less toxic metabolites by decomposing the group responsible for the toxicity of the parent compound. Taken together, the results of the chemical inhibitors, monoclonal antibodies, and cDNA-expressed CYP enzymes experiments demonstrated that CYP3A4 and CYP3A5 have essential functions in the metabolism of BAC, BMA, and BHA.

## 1. Introduction

*Aconitum* has been used as an essential drug in China and in other East Asian countries for centuries owing to its extremely excellent effect against rheumatosis and rheumatoid arthritis [1, 2]. It was reported that among 500 known prescriptions, the frequency of use of the *Aconitum *is 13.20%, ranked 9 [3]. Nevertheless, *Aconitum* is a toxic herb that can cause fatal cardiac poisoning [4–6] and is reportedly involved in suicidal and homicidal attempts [6, 7]. Since the therapeutic dose of *Aconitum* is very close to its toxic dose, its poisoning incidents are not uncommon. According to the literature data, in 188 cases of patients with *Aconitum*, 111 patients had varying degrees of toxic reactions [3]. The toxicological mechanism of *Aconitum* is widely recognized to be associated with voltage-dependent Na^+^ channels [8]. So the death of patients suffering from *Aconitum *toxicities always is cardiac arrest or respiratory insufficiency. Up to now, there is no effective antidote to *Aconitum* intoxication. The initial toxic signs are gastrointestinal including nausea, vomiting, and diarrhea. This is followed by a sensation of burning, tingling, and numbness in the mouth and face, and of burning in the abdomen [9, 10]. 

Therefore, prevention of *Aconitum* toxicity is a key issue for its safe application. Efflux transporters and metabolic enzymes act as the human body's first line of protection by limiting the invasion of toxicants [11]. Investigation on the roles of efflux transporters and metabolic enzymes in *Aconitum* is meaningful. 

*Aconitum* contains high amounts of three highly toxic diester-diterpene alkaloids (DDAs), namely, aconitine (AC), mesaconitine (MA), and hypaconitine (HA) [12]. Selling unprocessed *Aconitum* is forbidden on the Chinese market. With proper processing, AC, MA, and HA become exposed to abundant hydrolysis, transforming them into less toxic monoester-diterpene alkaloids (MDAs), namely, benzoylaconine (BAC), benzoylmesaconine (BMA), and benzoylhypaconine (BHA) [12]. 

MDAs are the primary active and toxic constituents of processed *Aconitum*. The alkaloid content in processed *Aconitum* is reportedly as follows: BMA > HA > BAC > MA > AC⁡ [13]. Aside from DDAs, MDAs are detectable in human serum and urine after oral intake of *Aconitum* extract [14]. Moreover, MDAs are evident in the plasma, liver, and kidney after the administration of DDAs, indicating that DDAs are chemically and biologically hydrolyzed into MDAs [2, 15]. BMA could reportedly recover the impaired resistance of thermally injured mice infected by HSV type 1 or *Candida albicans *[16]. The toxicity of MDAs is much less than those of DDAs (the human lethal dose is 1 mg to 4 mg) [17, 18]. However, the toxic reaction will still occur in the event of excessive MDA absorption [12]. Frequent cases of poisoning after the intake of processed *Aconitum* are encountered in clinics.

Previous studies suggested that DDAs could be metabolized into less toxic products by CYP enzymes (the major function of CYP3A) in the human liver microsomes [19–21]. The main metabolic pathways of DDAs include demethylation, dehydrogenation, hydroxylation, and didemethylation [19–21]. Efflux transporters such as P-glycoprotein, breast cancer resistance protein, and multidrug resistance-associated protein 2 were involved in the transport of DDAs [11]. Thus, the toxicity of DDAs is reduced by the effect of metabolic enzymes and efflux transporters [11, 19–21]. We investigated the role of efflux transporters in the transport of MDAs and found that efflux transporters did not mediate their transport [11]. This result indicates that the invasion of MDAs could not be limited by efflux transporters. Efflux transporters and metabolic enzymes act as the human body's first line of protection by limiting the invasion of toxicants [11]. However, thus far, the effect of metabolic enzymes on MDAs is unknown. Therefore, investigating the metabolic mechanism of MDAs for clinical practice is significant.

We tested phase I and phase II metabolism of MDAs (BAC, BMA, and BHA) by human liver microsomes in preliminary experiments. No phase II metabolites but several oxidative metabolites of BAC, BMA, or BHA were detected in the reaction systems. This study aims to identify the CYP metabolites of BAC, BMA, and BHA in human liver microsomes as well as the CYP isozymes responsible for their metabolism. High-resolution mass spectrometry was used to characterize the structures of their metabolites. Moreover, chemical inhibitors of specific CYP enzymes, CYP-specific inhibitory monoclonal antibodies, and cDNA-expressed CYP enzymes (CYP1A2, 2C8, 2C9, 2C19, 2D6, 2E1, 3A4, and 3A5) were used to verify which isozymes mediate in the metabolism of the CYP metabolites. These results on MDAs metabolism provide important data for the safe clinical use of processed *Aconitum*.

## 2. Materials and Methods

### 2.1. Materials

BAC, BMA, BHA, and testosterone (as internal standard) (purity > 98%) were purchased from Nanjing Zelang Medical Technology Co., Ltd. (Nanjing, China). Pooled human liver microsomes (HLMs, 20 mg/mL, including reductase), *β*-nicotinamide adenine dinucleotide phosphate (NADP), glucose-6-phosphate (6-P-G), and glucose-6-phosphate dehydrogenase (PDH) were purchased from BD Gentest Corp. (Woburn, MA, USA). cDNA-expressed CYP enzymes (CYP1A2, 2C8, 2C9, 2C19, 2D6, 2E1, 3A4, and 3A5), chemical inhibitors (fluvoxamine maleate, quercetin, amiodarone, omeprazole, quinidine, diethyldithiocarbamic acid, and ketoconazole) [22–27], and inhibitory monoclonal antibodies were also obtained from BD Gentest Corp. (Woburn, MA, USA). The monoclonal antibodies (mAbs, 10 mg/mL protein content) used were mAb-1A2 (anti-human CYP1A2), mAb-2C8 (anti-human CYP2C8), mAb-2C19 (anti-human CYP2C19), mAb-2D6 (anti-human CYP2D6), mAb-2E1 (anti-human CYP2E1), and mAb-3A4/5 (anti-human CYP3A4/5). Other chemicals, generally of reagent grade or better, were used as received.

### 2.2. Phase I Metabolism of BAC, BMA, and BHA in HLMs

A typical incubation system contained potassium phosphate (50 mM, pH 7.4), NADP (1.55 mM), 6-P-G (3.3 mM), MgCl_2_ (3.3 mM), PDH (0.4 U/mL), HLMs (0.4 mg protein/mL), and BAC, BMA, or BHA (10 *μ*M) in a total volume of 500 *μ*L. After a 5 min preincubation at 37°C, the reactions were started by adding NADPH [28]. Incubations were carried out for 90 min in a shaking water bath (150 rpm) at 37°C and were stopped by the addition of 4 mL ice-cold dichloromethane and 100 *μ*L testosterone (200 nM, used as an internal standard). The parent drug and its metabolites were extracted by vortexing for 8 min, after which they were centrifuged for 15 min at 1000 rpm. The supernatant was drawn into another tube and evaporated to dryness. The residue was dissolved with 50% methanol in water (125 *μ*L) and analyzed by liquid chromatography with tandem mass spectrometry detection (LC-MS/MS). Control incubations were conducted in the absence of a NADPH-regenerating system. 

To determine the protein concentration-dependent specificity of the enzyme, HLMs at concentrations of 0.2, 0.4, and 0.8 mg/mL were incubated at 37°C for 90 min in the above-described reaction system, respectively. To determine the time-dependent specificity, the reactions were processed for 30, 60, 90, and 120 min at 37°C in the reaction system, respectively. To determine the substrate concentration-dependent specificity, substrates at concentrations of 2.5, 5, 10, 15, and 20 *μ*M were incubated in HLMs at 37°C for 120 min, respectively. Each experiment was performed using three samples.

### 2.3. Effect of Chemical Inhibitors on the Metabolism of BAC, BMA, and BHA in HLMs

The selective inhibitors were chosen based on FDA recommendation, and previous reports, and are listed as follows: fluvoxamine maleate for CYP1A2, quercetin for CYP2C8, amiodarone for CYP2C9, omeprazole for CYP2C19, quinidine for CYP2D6, diethyldithiocarbamic acid for CYP2E1 and ketoconazole for CYP3A4/5 [22–27]. To obtain the maximum possible inhibition for each of the isoforms, the final inhibitor concentrations in the reaction mixtures were much higher than the reported Ki values. The concentration of each inhibitor was 5 *μ*M. The inhibitors were preincubated with all incubation mixtures for 5 min at 37°C before the initiation of the reaction by the addition of BAC, BMA, or BHA [20]. The mixture was then incubated for 120 min at 37°C. A comparison was made between the incubations of samples with and without the inhibitors. All incubations were carried out in triplicate.

### 2.4. Effect of mAbs on the Metabolism of BAC, BMA, and BHA in HLMs

Immunoinhibition studies were conducted by incubating HLMs with various amounts of human selective mAbs (mAb-1A2, mAb-2C8, mAb-2C19, mAb-2D6, mAb-2E1, and mAb-3A4/5). MAbs (10 *μ*L) were incubated with HLMs for 15 min on ice before the addition of the parent drug (BAC, BMA, or BHA, 10 *μ*M) and the NADPH-regenerating system [21]. The mixture was incubated at 37°C for 120 min. As a control, comparable incubations were performed with HLMs and Tris buffer (25 mM). All incubations were conducted in triplicate.

### 2.5. Phase I Metabolism of BAC, BMA, and BHA in cDNA-Expressed CYP Enzymes

The incubation procedures of BAC, BMA, or BHA in cDNA-expressed CYP enzymes were performed as described for HLMs, except that the concentration of the enzyme used was 40 pmol/mL [21, 29]. The isozymes used in this study included CYP 1A2, 2C8, 2C9, 2C19, 2D6, 2E1, 3A4, and 3A5. All incubations were performed in triplicate.

### 2.6. Identification and Quantitation of the Metabolites by LC-MS/MS

A quadrupole time of flight tandem mass spectrometer (Bruker, Daltonics, USA) was used to calculate the molecular weight of the metabolites, which could be deduced through their molecular formulas. The quantitative analysis procedure was as follows. The UPLC conditions were system, Waters Acquity; column, Acquity UPLC BEH C18 column (50 × 2.1 mm, 1.7 *μ*m, Waters, Milford, MA, USA); mobile phase A: 0.1% (v/v) formic acid in water; mobile phase B: 100% acetonitrile; gradient, 0 min to 0.5 min at 2 to 2% B, 0.5 min to 2 min at 2% to 20% B, 2 min to 4 min at 20% to 40% B, 4 min to 6.5 min at 40% to 70% B, and 6 min to 7 min at 70% to 2% B; flow rate, 0.35 mL/min; column temperature, 50°C; and injection volume, 10 *μ*L. The MS/MS detector used was a quadrupole tandem mass spectrometer (Waters, USA). Samples were analyzed using electrospray ionization in the positive model. The main working parameters were set as follows: capillary voltage, 3 kV; cone voltage, 50 V; collision voltage, 30 V; source temperature, 120°C; desolvation temperature, 500°C; desolvation gas flow, 600 L/Hr; cone gas flow, 50 L/Hr; and collision gas glow, 0.20 mL/min. Data were collected and analyzed by Waters Quantify software (Masslynx 4.1, Waters, USA). 

### 2.7. Data Analysis

Data are expressed as mean ± SD. One-way analysis of variance (ANOVA) with or without Tukey-Kramer multiple comparisons (post hoc) tests were used to evaluate statistical differences. Differences were considered significant when *P* values were less than 0.05.

## 3. Results

### 3.1. Identification of the BAC, BMA, and BHA Metabolites in HLMs

**Figure 1:**
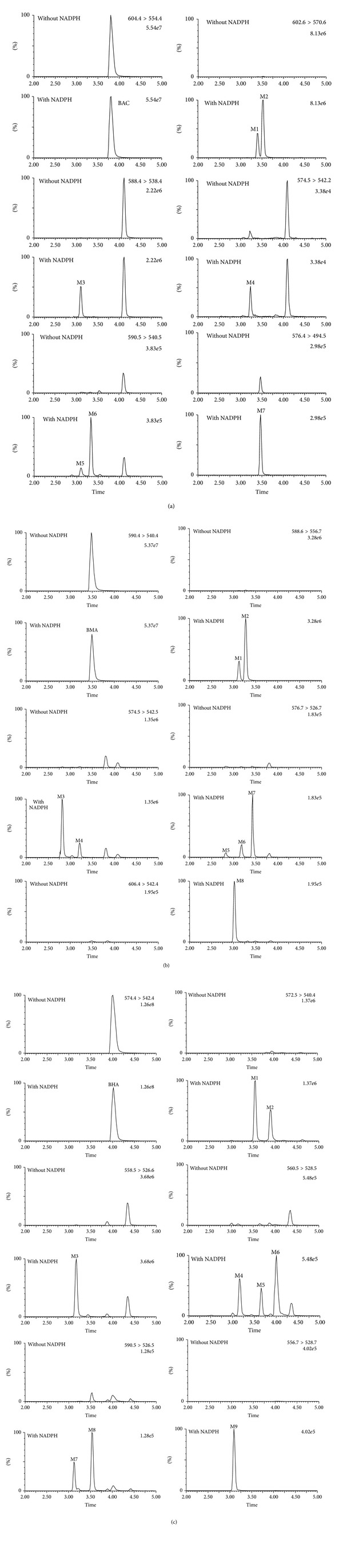
Representative selective ion current chromatograms of the BAC (a), BMA (b), and BHA (c) metabolites in HLMs incubated with 10 *μ*M of the parent compound for 120 min at 37°C. A total of 7, 8, and 9 metabolites were observed for BAC, BMA, and BHA after incubation with HLMs and NADPH, respectively.

To determine the best conditions for incubation, we optimized the incubation time, protein concentration, and substrate concentration. According to the largest formation of metabolites, the final protein concentration was selected to be 0.4 mg/mL and the appropriate incubation time was 120 min. Meanwhile, for the substrate concentration-dependent study, the formations of BAC, BMA, and BHA metabolites were all linear at concentrations of 2.5 *μ*M to 20 *μ*M, indicating that the concentration of 10 *μ*M BAC, BMA, or BHA that we chose for this study was suitable for the experiments. Compared with the negative control (without NADPH-regenerating system), a total of 7 BAC metabolites, 8 BMA metabolites and 9 BHA metabolites were found in the HLMs along with the NADPH-regenerating system (Figure 1). The metabolites were identified by the retention times, the chromatographic behaviors, and characteristic mass spectrometric fragmentation features, which are summarized in Table 1.

### 3.2. BAC Metabolites in HLMs

BAC, eluted at 3.77 min, possessed a pseudo-molecule ion [M+H]^+^ at *m/z* 604.3109, which corresponded to the smart molecular formula of C_32_H_45_NO_10_. The MS^2^ spectrum of [M+H]^+^ provided a number of characteristic fragment ions at *m/z* 586, 572, 554, 540, 522, and 508.

### 3.3. BAC Metabolites M1 and M2

M1 and M2 were eluted at 3.38 and 3.50 min, respectively. M1 and M2 showed a pseudo-molecule ion [M+H]^+^ at *m/z* 602.2951, which confirmed the smart molecular formula (C_32_H_43_NO_10_), indicating a loss of 2 Da (2H) from BAC, displaying that they were dehydrogenated metabolites of BAC. The MS^2^ spectrum of M1 and M2 showed fragmentation ions at *m/z* 584, 570, 552, 538, and 506, which were also 2 Da lower than the characteristic fragment ions of BAC. Thus, M1 and M2 metabolites were identified as dehydrogenation-BAC. H_2_ group could be taken off from positions 2, 12, and 15. Therefore, the exact positions of dehydrogenation were not confirmed.

### 3.4. BAC Metabolite M3

M3 had a retention time of 3.08 min, and its pseudo-molecule ion [M+H]^+^ was at *m/z* 588.2791. Thus, the smart molecular formula was calculated to be C_31_H_41_NO_10_, which had an observed loss of CH_4_ group compared with BAC. An MS/MS scan of M3 generated fragment ions at *m/z* 570, 556, 538, 524, 506, and 492, all of which were 16 Da lower than the fragment ions of the parent molecule. Therefore, M3 was identified as demethylated-dehydrogenated BAC metabolite. Metabolism may have occurred either by demethylation and then dehydrogenation or by dehydrogenation and then demethylation. Another dubious peak existed in the same reaction channel, which was eluted at 4.10 min. Its smart molecular formula was calculated to be C_32_H_45_NO_9_, which did not have similar structure with BAC. Meanwhile, this peak could not only be detected with NADPH-regenerating system, but also be detected without NADPH-regenerating system (negative control). Therefore, the peak eluted at 4.10 min was not the CYP metabolite of BAC. We speculated that it was just the endogenous substance of the phase I metabolic system.

### 3.5. BAC Metabolite M4

The M4 peak eluted at 3.20 min exhibited a pseudo-molecule ion [M+H]^+^ at *m/z* 574.2791 (C_30_H_39_NO_10_), which was 30 Da (C_2_H_6_) lower than that of BAC, confirming the smart molecular formula. The MS^2^ spectrum of M4 displayed fragment ions at *m/z* 542, 524, 510, 492, and 478, and these fragment ions were all 30 Da less than the corresponding fragments generated by BAC. Therefore, M4 was identified as didemethyl-dehydrogen-BAC or deethyl-dehydrogen-BAC. By the way, the peak having a retention time of 4.10 min was not the CYP metabolite of BAC, because of the peak could not only be detected with NADPH-regenerating system, but also be detected without NADPH-regenerating system (negative control). 

### 3.6. BAC Metabolites M5 and M6

UPLC retention times of 3.08 and 3.31 min were obtained for M5 and M6, respectively. Their pseudo-molecule ion [M+H]^+^ was both at *m/z* 590.2940, and the smart molecular formula was concluded to be C_31_H_43_NO_10_, which eliminated a CH_2_ group from the parent compound. The MS^2^ spectrum of fragment ions was seen at *m/z* 572, 558, 540, 526 and 508, which were 14 Da lower than the fragment ions of BAC. Conclusively, M5 and M6 were identified as demethyl-BAC, but the demethylation would occur at positions 1, 6, 16, and 18. Similarly, the peak eluted at 4.1 min was not the CYP metabolite of BAC, because the peak could not only be detected with NADPH-regenerating system, but also be detected without NADPH-regenerating system (negative control). 

### 3.7. BAC Metabolite M7

M7 exhibited a retention time of 3.45 min and had an [M+H]^+^ at *m/z* 576.2805. The smart molecular formula was confirmed to be C_30_H_41_NO_10_. The diagnostic ions at *m/z* 558, 544, 526, 512, 494, and 480 were 28 Da (C_2_H_4_) lower than the fragment ions in the MS^2^ spectra of BAC. Taken together, the results suggested that M7 may either be a metabolite of BAC with a loss of ethylation on the nitrogen atom or a didemethyl-BAC.

### 3.8. BMA Metabolites in HLMs

The peak elution at 3.48 min corresponded to BMA. BMA exhibited a pseudo-molecule ion [M+H]^+^ at *m/z* 590.2963 in the full-scan mass spectrum. The smart molecular formula of C_31_H_43_NO_10_ was demonstrated, and the MS^2^ spectrum of [M+H]^+^ showed fragment ions at *m/z* 572, 558, 540, 526, and 508.

### 3.9. BMA Metabolites M1 and M2

M1 and M2 were eluted at 3.11 and 3.27 min, respectively. They showed a pseudo-molecule ion [M+H]^+^ at *m/z* 588.2802, confirming the smart molecular formula of C_31_H_41_NO_10_ and a loss of 2 Da (2H) from BMA. The MS^2^ spectrum of M1 and M2 showed fragmentation ions at *m/z* 570, 556, 538, and 524, which were also 2 Da lower than the corresponding fragments generated by BMA. Thus, they are dehydrogenated metabolites of BMA. A loss of H_2_ group could occur in positions 2, 12, and 15, suggesting that the exact positions of dehydrogenation were not confirmed.

### 3.10. BMA Metabolites M3 and M4

The UPLC retention times of metabolites M3 and M4 were 2.81 and 3.20 min, and their pseudo-molecule ion [M+H]^+^ was both at *m/z* 574.2642. Their smart molecular formula was suggested to be C_30_H_39_NO_10_, and a CH_4_ group was lost compared with BMA. MS/MS scan of M3 and M4 showed fragment ions at *m/z* 556, 542, 524, 510, and 492, which were 16 Da lower than that of BMA. Therefore, M3 and M4 were identified as demethyl-dehydrogen-BMA. To speak of, the peaks eluted at 3.9 and 4.1 min were not the CYP metabolite of BMA, because these peaks could be detected with or without NADPH-regenerating system (negative control).

### 3.11. BMA Metabolites M5, M6, and M7

UPLC retention times of 2.82, 3.16, and 3.41 min were observed for M5, M6, and M7, respectively. Their pseudo-molecule ion [M+H]^+^ was all at *m/z* 576.2792, and their smart molecular formula was concluded as C_30_H_41_NO_10_, which lacked a CH_2_ group from the parent compound. The MS^2^ spectrum of fragment ions was seen at *m/z* 558, 544, 526, 512, and 494, which were 14 Da lower than the fragment ions of BMA. Therefore, they were estimated to be demethyl-BMA. 

### 3.12. BMA Metabolite M8

M8 had a retention time of 3.01 min and showed a pseudo-molecule ion [M+H]^+^ at *m/z* 606.2902. The smart molecular formula was calculated to be C_31_H_43_NO_11_. The MS^2^ spectrum of [M+H]^+^ provided a number of characteristic fragment ions at *m/z* 588, 574, 556, 542, and 524, which were 16 Da (O) higher than those of BMA. M8 was thus identified as hydroxyl-BMA.

### 3.13. BHA Metabolites in HLMs

BHA exhibited a retention time of 3.99 min, and its pseudo-molecule ion [M+H]^+^ appeared at *m/z* 574.3008, whose smart molecular formula was calculated to be C_31_H_43_NO_9_. The MS^2^ spectrum of [M+H]^+^ provided a number of fragment ions at *m/z* 558, 542, 524, 510, 492, and 478.

### 3.14. BHA Metabolites M1 and M2

M1 and M2 were eluted at retention times of 3.53 and 3.90 min, respectively. They both showed a molecule ion at *m/z* 572.2862 and a loss of H_2_ group from BHA. The MS^2^ scan of the two metabolites included major fragmentation ions at *m/z* 556, 540, 522, 508, 490, and 476, which were 2 Da lower than those of BHA. These results indicated that they were dehydrogen-BHA. Similar to BAC and BMA, the exact positions of dehydrogenation of BHA were not determined.

### 3.15. BHA Metabolite M3

M3 had a retention time of 3.16 min, and its pseudo-molecule ion [M+H]^+^ was at *m/z* 558.2709. The smart molecular formula was calculated to be C_30_H_39_NO_9_, and a loss of CH_4_ group was observed compared with BHA. MS/MS scan of M3 included fragment ions at *m/z* 542, 526, 508, and 494, which were 16 Da lower than the fragment ions of the parent compound. Therefore, M3 was identified as demethyl-dehydrogen-BHA. To speak of, because the peak could be detected with or without NADPH-regenerating system (negative control), the peak having a retention time of 4.3 min was not the CYP metabolite of BHA. 

### 3.16. BHA Metabolites M4, M5, and M6

The retention times of M4, M5, and M6 were 3.16, 3.63, and 3.99 min, respectively. Their pseudo-molecule ion [M+H]^+^ was found at *m/z* 560.2855, suggesting that their molecular formula was C_30_H_41_NO_9_, which is 14 Da (CH_2_) less than that of BHA. These results indicated that M4, M5, and M6 were demethylated metabolites of BHA. 

### 3.17. BHA Metabolites M7 and M8

M7 and M8 had UPLC retention times of 3.12 and 3.51 min, respectively. They showed a pseudo-molecule ion [M+H]^+^ at *m/z* 590.2978. The smart molecular formula was determined to be C_31_H_43_NO_10_, with an additional O group compared with the parent compound. The MS^2^ spectrum of [M+H]^+^ provided a number of characteristic fragment ions at *m/z* 574, 558, 540, 526, and 508, which were 16 Da higher than those of BHA. M7 and M8 were thus identified as hydroxyl-BMA. M8 was confirmed as BMA using the BMA standard.

### 3.18. BHA Metabolite M9

Metabolite M9 was eluted at 3.08 min and showed a pseudo-molecule ion [M+H]^+^ at *m/z* 556.2547. The smart molecular formula was confirmed to be C_30_H_37_NO_9_, which exhibited a loss of a CH_6_ group compared with the parent compound. MS/MS scan of M9 generated fragment ions at *m/z* 542, 528, 524, 497, 465, and 450. Therefore, M9 may be the demethylated-didehydrogenated metabolite of BHA.

### 3.19. The Effect of Chemical Inhibitors on the Metabolism of BAC, BMA, and BHA in HLMs

Based on the results, we learned that BAC, BMA, and BHA were metabolized into several CYP-mediated metabolites in HLMs together with the NADPH-regenerating system. To investigate which CYP isoform is responsible for their metabolism, various CYP enzyme chemical inhibitors were selected in this study. 

**Figure 2:**
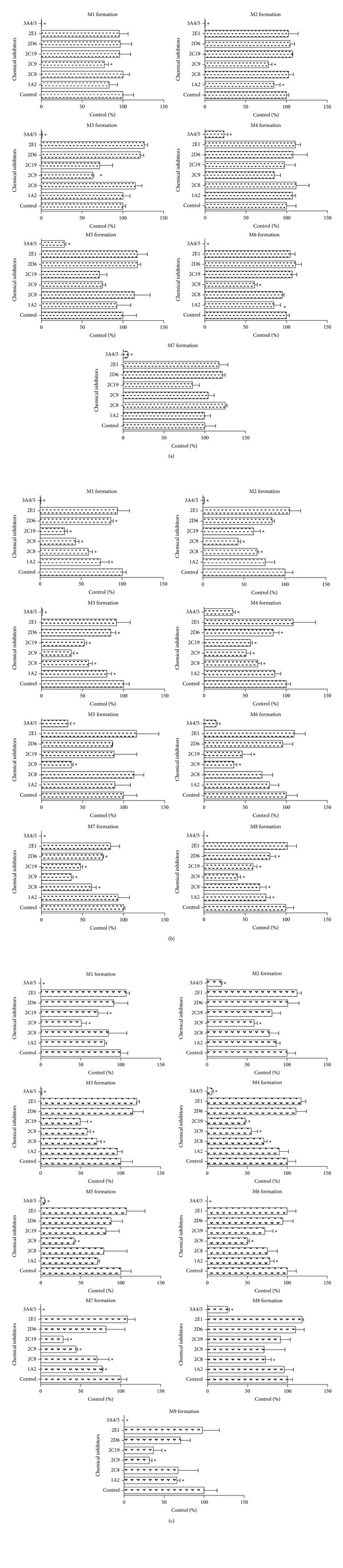
Effects of CYP enzyme chemical inhibitors on BAC (a), BMA (b), and BHA (c) metabolism. BAC, BMA, or BHA (10 *μ*M) was incubated with HLMs and NADPH-regenerating system in the presence of 5 *μ*M chemical inhibitors for 120 min at 37°C. In the control experiments, equivalent amounts of solvent were used in place of the inhibitors. Each column represents the mean of three formation rate determinations for the metabolites, and the error bars represent the standard deviation of the mean. Statistically significant differences compared with the control (*P* < 0.05) are marked with asterisks (∗).

For BAC (Figure 2(a)), ketoconazole (CYP3A4/5 inhibitor) produced a remarkable effect on the BAC metabolism. The formation of metabolites decreased by at least 70% compared with that of the control. Moreover, amiodarone (CYP2C9 inhibitor) had a less significant function in the formation of metabolites M1, M2, M3, and M6. Fluvoxamine maleate (CYP1A2 inhibitor) also had a minor effect on M2 and M6. However, the other inhibitors did not inhibit the formation of these seven metabolites. Taken together, CYP3A4/5 exhibited a major contribution to BAC metabolism. 

For BMA (Figure 2(b)), compared to the control group, ketoconazole greatly inhibited more than 65% of the formation of all BMA metabolites. All of the BMA metabolites were also significantly inhibited by the CYP 2C9 inhibitor. The production of M1, M2, M3, M4, M7, and M8 was decreased by the inhibitors of CYP2C8 and CYP2C19. Inhibitory effects on M1, M3, and M8 were observed using fluvoxamine maleate (CYP1A2 inhibitor). Quinidine (CYP2D6 inhibitor) had a negligible function in M1, M3, M4, M7, and M8. Diethyldithiocarbamic acid (CYP2E1 inhibitor) did not exhibit obvious inhibition on all eight metabolites. In general, BMA metabolism was predominantly mediated by CYP 3A, followed by CYP2C9 and CYP2C19.

For BHA (Figure 2(c)), ketoconazole strongly inhibited the formation of BHA metabolites by more than 75% compared with the control samples, suggesting that CYP3A4/5 was the major metabolic isozyme for BHA metabolism. The formation of BHA metabolites was significantly inhibited by amiodarone (CYP2C9 inhibitor). Simultaneously, quercetin (CYP2C8 inhibitor) notably reduced the formation of M3, M4, M7, and M8. Omeprazole (CYP2C19 inhibitor) contributed to the formation of M1, M3, M4, M6, M7, and M9. Moreover, the production of M6, M7, and M9 was influenced by fluvoxamine maleate (CYP1A2 inhibitor). According to the results, although several isozymes were involved in BHA metabolism, CYP3A4/5 was still the primary isozyme.

### 3.20. The Effect of mAbs on Metabolism of BAC, BMA, and BHA in HLMs

**Figure 3:**
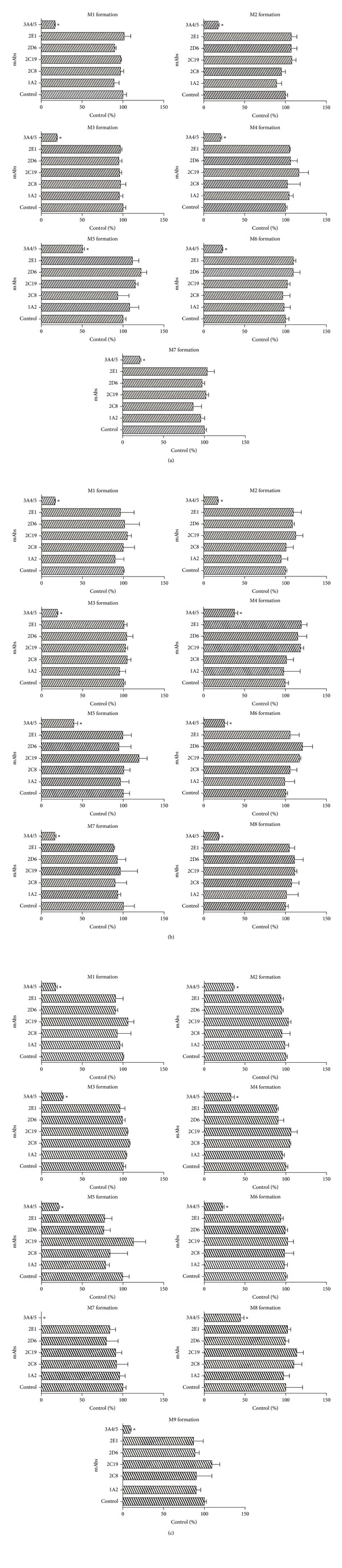
Effects of CYP enzyme mAbs on BAC (a), BMA (b), and BHA (c) metabolism. BAC, BMA, or BHA (10 *μ*M) was incubated with HLMs and NADPH-regenerating system in the presence of various individual mAbs for 120 min at 37°C. Equivalent amounts of Tris buffer were used instead of mAbs in the control experiments. Each column represents the mean of three formation rate determinations for the metabolites, and the error bars represent standard deviation of the mean. Statistically significant differences compared with the control (*P* < 0.05) are marked with asterisks (∗).

Percentage inhibition due to chemical inhibitors is never 100% and that selectivity is not always significant. Thus, in the next experiment, we used mAbs to further confirm which CYP isozymes mainly contribute to the metabolism of BAC, BMA, and BHA. We added mAbs 15 min prior to NADPH addition to start the reaction and tried to observe the effect of the possible loss of a particular CYP enzyme on the formation of each of the metabolites. As expected, CYP3A4/5 still had the most significant effect and maximally inhibited the metabolite formation compared with the rest of the mAbs (Figure 3). Other mAbs, such as mAb-1A2, mAb-2C8, mAb-2C9, mAb-2C19, mAb-2D6, and mAb-2E1, had no obvious inhibitory effect on the metabolic transformation of BAC, BMA, and BHA. CYP3A4/5 had an extremely important function in the metabolism of BAC, BMA, and BHA. 

### 3.21. Phase I Metabolism of BAC, BMA, and BHA in cDNA-Expressed CYP Enzymes

**Figure 4:**
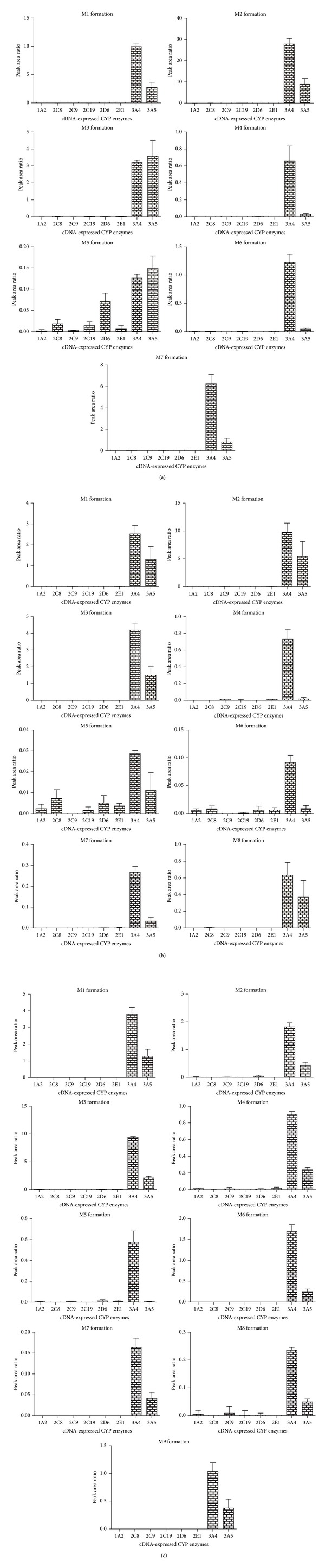
BAC (a), BMA (b), and BHA (c) metabolism in cDNA-expressed CYP enzymes. BAC, BMA, or BHA (10 *μ*M) was incubated with cDNA-expressed CYP enzymes (CYP 1A2, 2C8, 2C9, 2C19, 2D6, 2E1, 3A4, and 3A5) in the presence of NADPH-regenerating system for 120 min for 37°C. Data are expressed as the mean of three determinations, and the error bars represent standard deviation of the mean.

In the next experiments, rather than inhibiting the CYPs, we tried to incubate the compounds with specific cDNA-expressed CYPs bought from BD Life Sciences to reconfirm our chemical inhibitor and monoclonal antibody data. As anticipated, CYP3A4 still had the most significant effect, and 3A5 showed high formation rates for all the metabolites of BAC, BMA, and BHA (Figure 4). And the results indicated that the rate of BAC, BMA and BHA metabolism by CYP 3A4 was 32.96% ± 3.51%, 19.48% ± 2.02%, and 29.81% ± 3.54%, respectively.

## 4. Discussion

The Chinese medicine *Aconitum* as toxic drug is commonly used to treat rheumatism and cardiovascular diseases. In addition to efficacy, adverse events of *Aconitum* poisoning often occur in clinic. Prevention *Aconitum* toxicity is a key issue for its safe application. Efflux transporters and drug-metabolizing enzymes protect the human body by functioning as the important defense line that limits the invasion of toxicants. In the present study, we have demonstrated that monoester-diterpene alkaloids (MDAs) including BAC, BMA, and BHA were metabolized into several metabolites in HLMs. And CYP3A4 and CYP3A5 were the predominant isozymes responsible for their metabolism by the experiments of chemical inhibitors, mAbs and cDNA-expressed CYP enzymes. A total of 7, 8, and 9 metabolites were found, respectively, when BAC, BMA, or BHA was incubated in the HLMs along with NADPH-regenerating system. A majority of metabolite reaction channels had at least two metabolites, but they did not characterize the exact position of transformation on account of several methyl and hydroxyl groups in the structure of MDAs. What is more, the structures of MDAs are so complex and contain so many similar moieties that it would be extremely difficult to unambiguously identify metabolites solely using mass spectrometry. NMR data would be ideal for such compounds, but it may be difficult to generate sufficient amounts of metabolites. For these reasons, the metabolite characterization of the present study is rather preliminary and speculative, but it may be helpful to understand the metabolic profile of MDAs.

BAC, BMA, and BHA are the main, active and toxic constituents of processed *Aconitum. *The unprocessed *Aconitum* is prohibited to sell in the market. Even though the toxicity of BAC, BMA, and BHA is much less than diester diterpene alkaloids [12], attention is still need. The toxic reaction will still occur by excessive absorption of BAC, BMA, and BHA. As is well known, efflux transporters and metabolic enzymes act as the first line to protect the body by limiting the invasion of toxicants. Previous studies indicated that the efflux transporters including P-gp, BCRP, and MRP2 did not mediate the transport of BAC, BMA, and BHA, suggesting that the invasion of BAC, BMA, and BHA could not be limited by efflux transporters [11]. In this case, the role of metabolic enzymes in BAC, BMA, and BHA is extremely important. 

**Figure 5:**
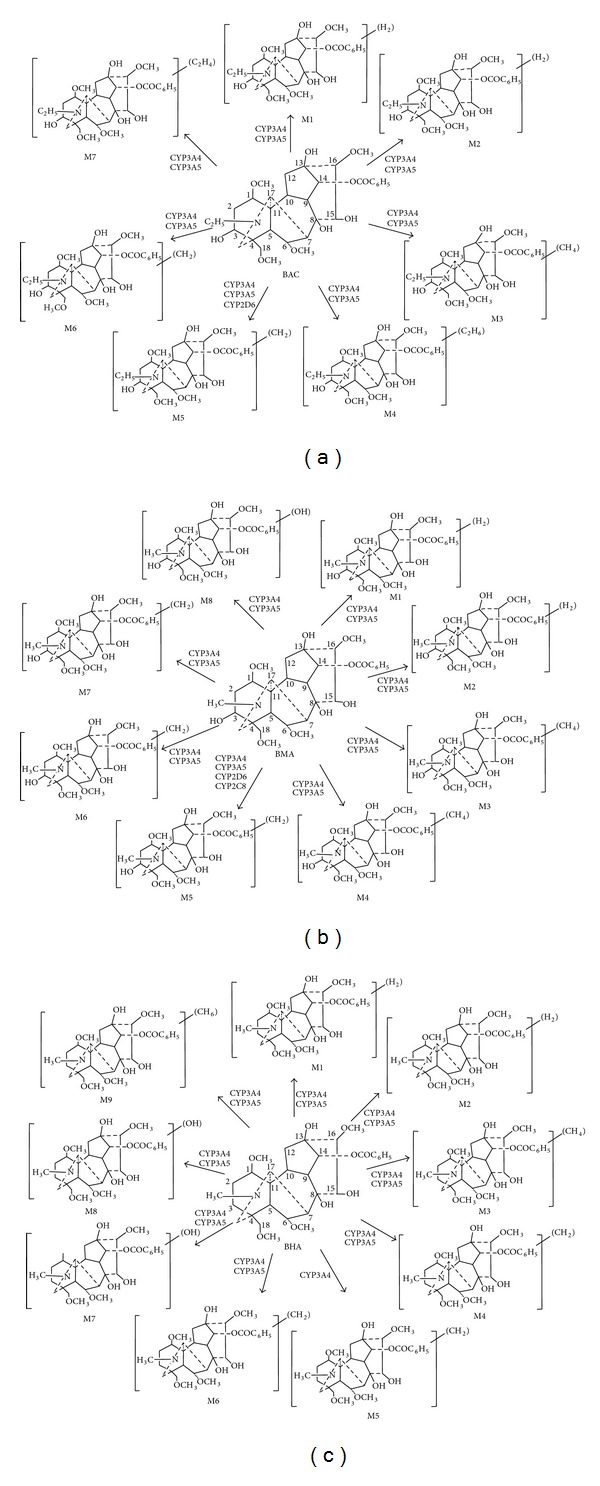
Proposed metabolic pathways of BAC (a), BMA (b), and BHA (c) in HLMs and CYP isozymes involved in their metabolism.

We investigated the metabolism of BAC, BMA, and BHA for the first time. A total of 7, 8, 9 metabolites were found for BAC, BMA, and BHA in HLMs with NADPH-regenerating system (Figure 1). BAC, BMA, and BHA have very similar chemical structures. A methyl moiety attaches to the nitrogen atom and a hydroxyl group links to position C3 in the structure of MA, whereas the unit attached to the nitrogen is ethyl in the BAC structure. The group linked to the nitrogen atom in the structure of BHA is methyl, but the hydroxyl moiety at position C3 is not observed. Therefore, their metabolic pathways were also similar, including demethylation, dehydrogenation, demethylation-dehydrogenation, hydroxylation, and didemethylation (Figure 5). What is more, these metabolic pathways were similar to those of DDAs [19–21]. Previous studies indicated that HA could be metabolized into MA by the effect of CYP enzymes. As expected, BHA was also metabolized into BMA in the present study. It was reported that the high toxicity of *Aconitum* alkaloids was due to the acetyl group at C8, the hydroxyl group at C13, four methoxyl groups at C1, C6, C16, and C18, and the benzoyl ester group at C14 [9], meaning that the loss of these groups will decrease the compound's toxicity. According to our results, all the metabolites (except for the hydroxylation metabolite) were either loss of methyl from methoxyl group or dehydrogenation from hydroxyl group, indicating that their toxicology was less than that of the parent compound. 

The results showed that CYP enzymes mediated the metabolism of BAC, BMA, and BHA, reducing the accumulation of toxic parent compound in the body. In the next experiments, we further investigated which CYP isozymes are responsible for their metabolism. The targeted isozymes included CYP1A2, 2C8, 2C9, 2C19, 2D6, 2E1, 3A4, and 3A5, because up to 90% of human drug metabolism may be attributed to these eight enzymes. In the chemical inhibitors experiments, the major hepatic enzyme responsible for the metabolism of BAC, BMA, and BHA was identified as CYP3A4/5, while chemicals selective for CYP2C8, CYP2C9, and CYP2C19 made a minor effect on their formations. It was assumed that a selective chemical inhibitor raised against a human CYP isoform at high concentration, which was more than the Ki value, will cross-react with other CYP isozymes. For example, recent studies suggest that omeprazole is a substrate probe for CYP2C19, however in vitro studies show that omeprazole could be metabolized by CYP3A4 in the same way [30]. As is well known, the chemical inhibitors are never 100% and their selectivity is not always great. Hence, we used mAbs experimental approach to reconfirm our results. We demonstrated that antibody against human CYP3A4/5 was the most potent and inhibited the formation of BAC, BMA, and BHA. Furthermore, the experiment of cDNA-expressed CYP enzymes showed that CYP3A4 and CYP3A5 were the major isozymes for the metabolism of BAC, BMA, and BHA, and the BAC metabolite M5 generated by CYP2D6, and the BMA metabolites M5 and M6 generated by CYP2C8, 2D6, and 3E1 supported the minimal roles of these enzymes. However, the inhibition experiments of BAC, BMA, and BHA did not support that CYP2C8, 2D6, and 3E1 were the subordinate enzymes which mediated the biotransformation of BAC, BMA, and BHA. Taken together, the results of cDNA-expressed CYP enzymes, chemical inhibitors, and mAbs experiments, we come to the conclusion that CYP3A4 and CYP3A5 were the major isozymes responsible for the metabolism of BAC, BMA, and BHA (Figure 5).

CYP3A4 and CYP3A5 also contributed a great role to the metabolism of DDAs (AC, MA, and HA) [11, 20, 31]. DDAs could not be only chemically hydrolyzed into MDAs, but also biologically hydrolyzed [2, 15]. In other words, after the intake of processed *Aconitum, *three kinds of behavior will occur: DDAs transformed into less toxic metabolites by CYP3A4 and CYP3A5; MDAs transformed into less toxic metabolites by CYP3A4 and CYP3A5; and DDAs transformed into MDAs by the esterase, which will greatly reduce the toxicity of *Aconitum.* Therefore, protection or promotion of CYP3A function could prevent excessive *Aconitum* absorbed into the bloodstream, thereby avoiding toxic reactions.

CYP3A is one of the most important CYP isoform responsible for drug metabolism by humans, because it is highly expressed in critical tissues such as the gastrointestinal tract and liver, and it is involved in the oxidative biotransformation of numerous clinically useful therapeutic agents. However, many factors could regulate CYP3A expression and activity [32]. Accordingly, the potential for drug interactions with these drugs as well as other CYP3A substrates, when given concomitantly, is high. Metabolism involving CYP3A is also likely to be affected by liver disease as well as aging. Therefore, more attention should be paid when we ingest *Aconitum*, even though the CYP enzymes could act as a defense line to decrease its toxicity.

## Figures and Tables

**Table 1:** LC-MS^n^ data of the BAC, BMA, and BHA metabolites in HLMs with NADPH.

Metabolite	[M+H]^+^ (*m*/*z*)	Smart formula [M]	Major fragment ions	Identification
BAC	604.3109	C_32_H_45_NO_10_	586, 572, 554, 540, 522, 508	BAC
M1	602.2951	C_32_H_43_NO_10_	584, 570, 552, 538, 506	Dehydrogen-BAC
M2	602.2949	C_32_H_43_NO_10_	584, 570, 552, 538, 506	Dehydrogen-BAC
M3	588.2791	C_31_H_41_NO_10_	570, 556, 538, 524, 506, 492	Demethyl-dehydrogen-BAC
M4	574.2638	C_30_H_39_NO_10_	542, 524, 510, 492, 478.	Didemethyl-dehydrogen-BAC/Deethyl-dehydrogen-BAC
M5	590.2940	C_31_H_43_NO_10_	572, 558, 540, 526, 508	Demethyl-BAC
M6	590.2944	C_31_H_43_NO_10_	572, 558, 540, 526, 508	Demethyl-BAC
M7	576.2805	C_30_H_41_NO_10_	558, 544, 526, 512, 494, 480	Deethyl-BAC/didemethyl-BAC

BMA	590.2963	C_31_H_43_NO_10_	572, 558, 540, 526, 508	BMA
M1	588.2802	C_31_H_41_NO_10_	570, 556, 538, 524	Dehydrogen-BMA
M2	588.2808	C_31_H_41_NO_10_	570, 556, 538, 524	Dehydrogen-BMA
M3	574.2642	C_30_H_39_NO_10_	556, 542, 524, 510, 492	Demethyl-dehydrogen-BMA
M4	574.2647	C_30_H_39_NO_10_	556, 542, 524, 510, 492	Demethyl-dehydrogen-BMA
M5	576.2789	C_30_H_41_NO_10_	558, 544, 526, 512, 494	Demethyl-BMA
M6	576.2790	C_30_H_41_NO_10_	558, 544, 526, 512, 494	Demethyl-BMA
M7	576.2792	C_30_H_41_NO_10_	558, 544, 526, 512, 494	Demethyl-BMA
M8	606.2902	C_30_H_43_NO_11_	588, 574, 556, 542, 524	Hydroxyl-BMA

BHA	574.3008	C_31_H_43_NO_9_	558, 542, 524, 510, 492, 478	BHA
M1	572.2857	C_31_H_41_NO_9_	556, 540, 522, 508, 490, 476	Dehydrogen-BHA
M2	572.2862	C_31_H_41_NO_9_	556, 540, 522, 508, 490, 476	Dehydrogen-BHA
M3	558.2709	C_30_H_39_NO_9_	542, 526, 508, 494	Demethyl-dehydrogen-BHA
M4	560.2843	C_30_H_41_NO_9_	544, 528, 510, 496, 464	Demethyl-BHA
M5	560.2855	C_30_H_41_NO_9_	544, 528, 510, 496, 464	Demethyl-BHA
M6	560.2857	C_30_H_41_NO_9_	544, 528, 510, 496, 464	Demethyl-BHA
M7	590.2978	C_31_H_43_NO_10_	574, 558, 540, 526, 508	Hydroxyl-BHA
M8	590.2959	C_31_H_43_NO_10_	574, 558, 540, 526, 508	BMA
M9	556.2547	C_30_H_37_NO_9_	542, 528, 524, 497, 465, 450	Demethyl-didehydrogen-BHA

## Abbreviations

MDAs: Monoester-diterpene alkaloids
DDAs: Diester-diterpene alkaloids
AC: Aconitine
MA: Mesaconitine
HA: Hypaconitine
BAC: Benzoylaconine
BMA: Benzoylmesaconine
BHA: Benzoylhypaconine
HLMs: Human liver microsomes
mAbs: Monoclonal antibodies
NADP: *β*-nicotinamide adenine dinucleotide phosphate
6-P-G: Glucose-6-phosphate
PDH: Glucose-6-phosphate dehydrogenase
P-gp: P-glycoprotein
BCRP: Breast cancer resistance protein
MRP2: Multidrug resistance-associated protein 2
LC-MS/MS: Liquid chromatography-tandem mass spectrometry
CYP450: Cytochrome P450
Ki: Kinetic inhibition rate constants
UPLC: Ultraperformance Liquid-Chromtography
NMR: Nuclear magnetic resonance.
